# Results of Surgical Repair of Hilar Renal Artery Aneurysm to Preserve Renal Blood Flow

**DOI:** 10.3400/avd.oa.20-00020

**Published:** 2020-09-25

**Authors:** Takumi Kawase, Yosuke Inoue, Jiro Matsuo, Atsushi Omura, Yoshimasa Seike, Kyokun Uehara, Hiroaki Sasaki, Hitoshi Matsuda

**Affiliations:** 1 Department of Cardiovascular Surgery, National Cerebral and Cardiovascular Center

**Keywords:** open surgery, preservation, renal function, hilar, renal artery aneurysm

## Abstract

**Objective:** Surgical indications and procedures for hilar renal artery aneurysm (HRAA) are controversial in terms of invasiveness and feasibility. Catheter treatment is minimally invasive but leads to renal dysfunction due to renal infarction. This study aims to investigate the results of surgical repair of HRAA.

**Method:** Fourteen patients (58.7±11.6 years old, 7 male) who underwent surgical repair of HRAA were retrospectively reviewed. Nine patients (64%) developed HRAA in the right renal artery, and the mean maximum aneurysmal diameter was 25.9±10.3 mm. HRAA was exposed via the extraperitoneal approach. HRAA was resected completely, and reconstruction of renal arteries was performed by direct closure in two, direct anastomosis in nine, and interposition of saphenous vein graft in three patients.

**Results:** The average operation and renal ischemic times were 186±49 and 35±16 min, respectively. No operative death occurred, and postoperative renal function at the time of discharge had not deteriorated (creatinine, 0.74±0.15 mg/dl). During the follow-up periods (4.7±5.1 years), there was no death, no new introduction of hemodialysis, and no recurrence of renal artery aneurysm.

**Conclusion:** Surgical repair of HRAA remains a valid option because of its operative safety, preservation of renal function, and long-term feasibility and patency.

## Introduction

Recently, renal artery aneurysms are increasingly detected by chance with the progress of imaging diagnosis. Some reports indicate that renal artery aneurysm is slow to expand, and the frequency of rupture is 3%–5%.^1)^ However, the prognosis is still unknown because the incidence of renal artery aneurysm is reportedly as low as 0.1%.^1)^

When a renal artery aneurysm is located at the renal hilum, the surgical indications and procedures are more controversial in terms of invasiveness and feasibility. Noninvasive catheter intervention is one of the options to treat hilar renal artery aneurysm (HRAA).^2–5)^ However, complete preservation of the renal blood flow might be difficult after the embolization of HRAA.^2)^

To throw light on this issue, we have aggressively indicated the resection of HRAA followed by revascularization of renal arteries. This study aims to investigate the results of surgical repair of HRAA.

## Materials and Methods

Between March 2002 and February 2019, 14 patients (58.7±11.6 years old, 7 male) underwent surgical repair of HRAA (see **Table 1**). HRAA was first diagnosed by contrast medium-enhanced computed tomographic angiography (CTA) by chance without any specific symptoms, except for one patient who complained of back pain. The HRAA was located in the right renal artery in nine (64%) and the left in five (36%) patients. The maximum diameter of the HRAA was 25.9±10.3 mm, and calcified aneurysmal wall was observed in five patients. As for comorbidities, hypertension was observed in five and hyperlipidemia in four. Other detected aneurysms were cerebral artery aneurysm in one and splenic artery aneurysm in two. No patient presented preoperative renal dysfunction (creatinine, 0.70±0.14 mg/dl). Five patients (36%) had a surgical history of laparotomy for appendicitis, uterine myoma, Cesarean section, duodenal ulcer, and splenic rupture.

**Table 1:** Table 1 Patient demographics

Patient	Age/sex (y.o.)	Location	Diameter (mm)	Aneurysmal wall calcification	Preoperative creatinine (mg/dl)	History of laparotomy/other aneurysm	Skin incision	Procedure	Operation time (min)	Renal ischemic time (min)	Ringer’s solution	Pathological findings
1	72/M	Left	55	Total	0.71	Appendicitis	Pararectal	Direct repair	195	43	−	No data
2	61/F	Left	20	Total	0.53	Uterine myoma	Pararectal	Direct repair	170	27	−	No data
3	62/F	Right	15	Partial	0.67	Duodenal ulcer	Pararectal	Direct repair	145	35	−	No data
						Splenic artery aneurysm						
4	40/M	Right	41	Not calcified	0.92		Pararectal	Direct repair	132	31	−	No data
5	54/M	Right	20	Not calcified	0.65		Transverse	Aneurysmorrhaphy	197	25	+	True aneurysm
6	63/F	Right	20	Partial	0.64	Splenic artery aneurysm	Transverse	Aneurysmorrhaphy	127	27	+	True aneurysm (AA)
7	68/F	Right	28	Not calcified	0.58	Cesarean section	Hypochondriac	SVG interposition	269	56	+	True aneurysm (AA)
8	44/M	Right	25	Not calcified	0.67	Spleen rupture	Pararectal	SVG interposition	256	13	+	False aneurysm with disruption of internal elastic lamina
9	71/M	Right	20	Not calcified	0.84		Hypochondriac	Direct repair	198	40	−	True aneurysm (AA)
10	41/F	Left	25	Not calcified	0.62		Pararectal	Direct repair	161	28	−	True aneurysm
11	53/F	Right	20	Not calcified	0.55		Pararectal	Direct repair	140	30	+	True aneurysm
12	78/F	Left	23	Not calcified	0.67	Cerebral artery aneurysm	Pararectal	SVG interposition	232	25	−	True aneurysm
13	58/M	Right	28	Not calcified	0.95		Pararectal	Direct repair	246	76	+	True aneurysm with fragmentation of internal elastic lamina
14	57/M	Right	23	Total	0.93		Pararectal	Direct repair	141	35	+	True aneurysm (AA)

Pararectal: pararectal oblique incision; transverse: transverse incision; hypochondriac: hypochondriac transverse incision; direct repair: direct anastomosis of branch renal artery; SVG: saphenous vein graft; AA: atherosclerotic aneurysm

This retrospective observational study was approved by the institutional review board (M30-057), and because of its retrospective fashion, individual verbal and written informed consent was waived.

### Treatment strategy for renal artery aneurysm

Basically, the surgical repair of HRAA is indicated when the diameter is larger than 20 mm. However, according to the patient’s strong treatment preference, the endovascular embolization with coil was exceptionally indicated at the diameter of 15 mm only when the massive renal infarction could be avoided definitely after obtaining careful informed consent. When massive renal infarction is unavoidable, repair of HRAA is indicated after the enlargement more than 20 mm, and the HRAA smaller than 15 mm was treated with optimal medical treatment.

During the study period, surgical repair was the principal choice of treatment for HRAA. For 9 patients, however, endovascular embolization of HRAA was performed with the patients’ consent, and 25 patients were medically followed up due to smaller aneurysmal size (≤20 mm).

### Surgical procedure

**Figure 1:**
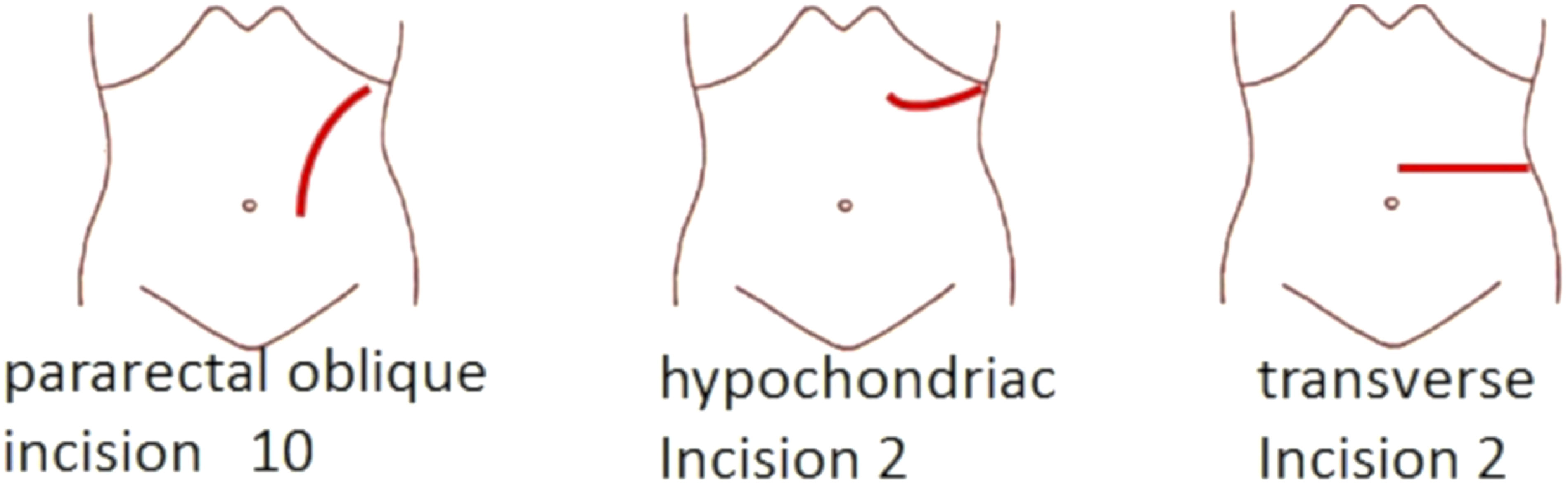
Fig. 1 Skin incisions for surgical repair of hilar renal artery aneurysm.

After the skin incision, a pararectal oblique incision in 10, hypochondriac transverse incision in 2, and transverse incision in 2 patients (**Fig. 1**) were made.

In the last five patients, we selected the pararectal oblique incision. HRAA was exposed via the extraperitoneal approach by dissecting para-peritoneal fat tissue and opening Gerota’s fascia. After systemic heparinization, renal arteries were clamped, and the aneurysm was resected completely. Cold Ringer’s solution (124±75 ml, 4°C) was infused directly to the peripheral renal arteries if renal ischemic time was expected to be longer than 40 min.^1,6,7)^

Renal artery reconstruction depended on the exact location of HRAA. In two patients, several small branches arose from aneurysm and were difficult to reconstruct individually. Thus, we selected aneurysmorrhaphy in a bid to preserving complete blood flow and reducing the aneurysmal diameters as much as possible to avoid rupture. In the other 12 patients, the renal artery was reconstructed by direct anastomosis in 9 and interposition of a saphenous vein graft in 3 patients (**Table 1**). Patency of the renal artery was confirmed by means of direct ultrasonography.

## Results

### Early results

The mean operation time was 186±49 min, and the mean renal ischemic time was 35±16 min. No patient needed blood transfusion. No operative death was encountered, and postoperative renal function at the time of discharge had not deteriorated (creatinine, 0.74±0.15 mg/dl). All patients were discharged home 11±4 days after the operation.

### Long-term results

During the follow-up periods (4.7±5.1 years), no death or new introduction of hemodialysis occurred, except for one patient with putaminal hemorrhage who was followed up without antiplatelet agents. Eight patients were followed up while being medicated with antiplatelet drugs. All patients were followed up with abdominal echo examinations, and no recurrence of renal artery aneurysm was noted.

### Representative case (patient 12)

**Figure 2:**
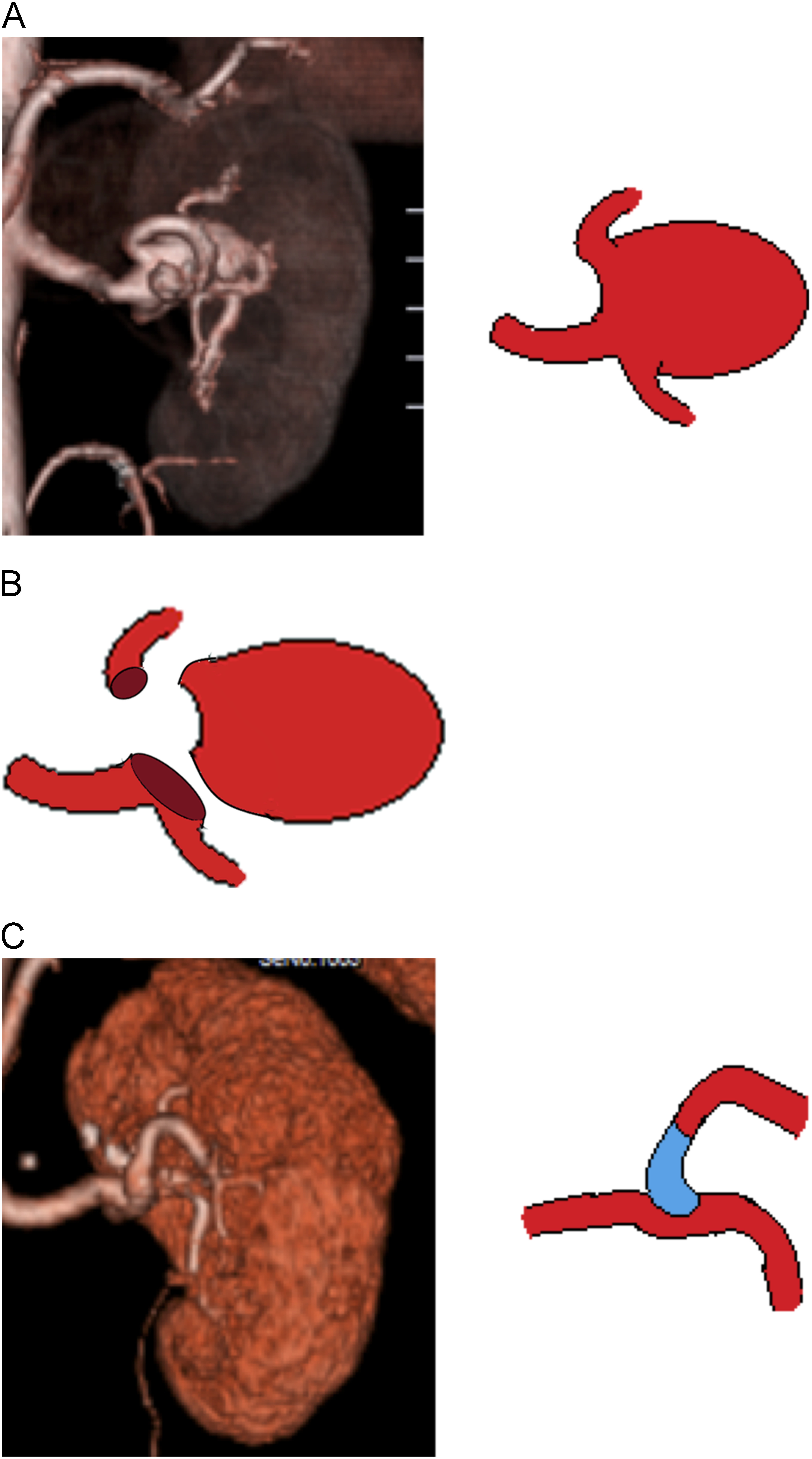
Fig. 2 (**A**) Preoperative computed tomographic angiography (CTA) of representative case of surgical repair of hilar renal artery aneurysm (patient 12). (**B**) Schema of intraoperative resected area in a representative case of surgical repair. (**C**) Postoperative CTA of representative case of surgical repair of hilar renal artery aneurysm (patient 12).

A 78-year-old woman with past medical history significant for hypertension, hyperlipidemia, transurethral bladder tumor resection for early stage bladder carcinoma, and 3-mm cerebral aneurysm at the left middle cerebral artery was referred for asymptomatic left HRAA with a diameter of 23 mm (**Fig. 2A**). Her serum creatinine level was 0.66 mg/dl. With the patient in the right semi-recumbent position, the HRAA was exposed behind Gerota’s fascia by means of a pararectal oblique incision and extraperitoneal approach with careful dissection of the renal veins. After systemic heparinization, the proximal portion of the left renal artery and the upper and lower segmental branches were clamped, and the HRAA, 2.5 cm in length, was resected (**Fig. 2B**). Continuity between the proximal portion and lower segmental branch was preserved, and the upper segmental branch was reconstructed with the interposition of a short saphenous vein graft by means of bilateral end-to-side anastomosis with 6-0 polypropylene continuous suture. Renal blood flow was confirmed by direct echo-Doppler imaging. The operation and left renal ischemic time were 233 and 25 min, respectively. Postoperative CTA showed no residual aneurysm and patent flow to left kidney (**Fig. 2C**). Postoperative creatinine level was 0.79 mg/dl. The patient was discharged home without any significant complications and with continuing antiplatelet and anticoagulation therapy with aspirin, clopidogrel, or warfarin.

## Discussion

Our study shows that the results of surgical repair of HRAA are acceptable because it was carried out without the need for blood transfusion and with no occurrence of operative death or deterioration of renal function. We could also confirm the long-term feasibility of complete resection of HRAA followed by surgical reconstruction of the renal blood flow.

As the incidence of rupture of renal artery aneurysms 20 mm or more in diameter has been reported as 3%–5%, the indication of repair for renal artery aneurysm is usually defined as 20 mm.^1,8)^ The 14 patients enrolled in this study had HRAAs with a mean diameter of 25.9±10.3 mm. Although the aneurysm of one patient (patient 3), was 15 mm in diameter, it was saccular in type with a noncalcified aneurysm wall, which has been reported as a risk factor for rupture.^6,9–11)^

Favorable results have been reported for endovascular treatment of renal aneurysm.^2–5)^ Zhang et al. performed endovascular repair for 15 patients with renal artery aneurysm or renal arteriovenous fistula and observed no periprocedural mortality or major complications with a technical success rate of 100%.^2)^ Tsilimparis et al. compared open surgery and endovascular repair and reported similar perioperative complication rates but shorter hospitalization, 2.3 days, after endovascular repair compared with 6.3 days after open repair.^3)^ Buck et al. reported a lower rate of postoperative complication and shorter length of stay after endovascular repair.^4)^

Endovascular repair of HRAA consists of coil embolization and stent grafting. Coil embolization is favorable for treating saccular aneurysm or aneurysm with a narrow neck, but it can be expensive and time-consuming depending on sac volume. When the neck of HRAA is wide, migration of the coil or residual sac perfusion can be expected, and stent grafting might be considered. However, due to the risk of thrombosis, stent grafting is reserved for a main renal artery with a ≧6-mm diameter.^2)^

The common complications of endovascular repair of HRAA include renal artery dissection, coil migration, and postembolization syndrome. In addition, the renal blood flow is often interrupted if the HRAA is not saccular, and concern remains about postoperative renal dysfunction.

Robinson et al. emphasized the advantage of open surgery repair in terms of renal function because it can preserve segmental branches.^9)^ They repaired 26 renal artery aneurysms of 24 patients. In vivo repair was performed for 22 patients, and ex vivo repair was required for 4 when renal artery aneurysm involved distal subsegmental branches abutting the kidney hilum. No death had occurred at 30 days, but morbidity was detected in 11.5% of patients. The renal function of those who underwent surgical repair was generally maintained. Long-term patency was 94%, and no rupture or recurrent aneurysm was observed during 99 months.

In our study, it was also found that, during the follow-up period of 4.7±5.1 years, no patient has suffered from renal dysfunction or rupture, suggesting that, for patients for whom open surgery is expected to be safe and whose renal function is normal, complete resection of HRAA followed by direct reconstruction of renal blood flow should be considered.

On the other hand, some reports have documented that patients with a renal artery aneurysm tended to have multiple visceral or peripheral aneurysms.^10)^ In our study, too, other aneurysms were detected by chance in two patients (14%). To obtain good prognostic results, following up not only for recurrence of renal artery aneurysms but also the development of other aneurysms seems to be critical.

## Limitations

This study has several limitations related to its non-comparative retrospective fashion and relatively small number of patients.

## Conclusion

Surgical repair of HRAA still remains a valid option because of its operative safety, preservation of renal function, and long-term feasibility and patency.

## References

[1] Coleman DM, Stanley JC. Renal artery aneurysms. J Vasc Surg 2015; 62: 779-85. 26213273 10.1016/j.jvs.2015.05.034

[2] Zhang Z, Yang M, Song L, et al. Endovascular treatment of renal artery aneurysms and renal arteriovenous fistulas. J Vasc Surg 2013; 57: 765-70. 23312837 10.1016/j.jvs.2012.09.042

[3] Tsilimparis N, Reeves JG, Dayama A, et al. Endovascular vs open repair of renal artery aneurysms: outcomes of repair and long-term renal function. J Am Coll Surg 2013; 217: 263-9. 23769185 10.1016/j.jamcollsurg.2013.03.021

[4] Buck DB, Curran T, McCallum JC, et al. Management and outcomes of isolated renal artery aneurysms in the endovascular era. J Vasc Surg 2016; 63: 77-81. 26386509 10.1016/j.jvs.2015.07.094 PMC4698072

[5] Tang S, Niu G, Fang D, et al. The diagnosis and endovascular therapy of renal artery aneurysm. Medicine (Baltimore) 2017; 96: e8615. 29381933 10.1097/MD.0000000000008615 PMC5708932

[6] English WP, Pearce JD, Craven TE, et al. Surgical management of renal artery aneurysm. J Vasc Surg 2004; 40: 53-60. 15218462 10.1016/j.jvs.2004.03.024

[7] Henke PK, Cardneau JD, Welling TH 3rd, et al. Renal artery aneurysm: a 35-year clinical experience with 252 aneurysms in 168 patients. Ann Surg 2001; 234: 454-62; discussion, 462-3. 11573039 10.1097/00000658-200110000-00005 PMC1422069

[8] Ikarashi J, Yamanaka K, Iwakura A. Surgical treatment of renal artery aneurysm. Jpn J Cardiovasc Surg 2018; 47: 307-11. (in Japanese）

[9] Robinson WP 3rd, Bafford R, Belkin M, et al. Favorable outcomes with in situ techniques for surgical repair of complex renal artery aneurysms. J Vasc Surg 2011; 53: 684-91. 21144690 10.1016/j.jvs.2010.10.050

[10] Hidai H, Kinoshita Y, Murayama T, et al. Renal artery aneurysm: clinical report and analysis on diagnosis and treatment. Jpn J Urol 1982; 73: 177-88. (in Japanese） 7098227

[11] Pfeiffer T, Reiher L, Grabitz K, et al. Reconstruction for renal artery aneurysm: operative techniques and long-term results. J Vasc Surg 2003; 37: 293-300. 12563198 10.1067/mva.2003.117

